# Use of Screws

**Published:** 1898-05

**Authors:** H. W. Arthur

**Affiliations:** Pittsburg.


ARTICLE V.

### USE OF SCREWS.

BY H. W. ARTHUR, D. D. S., PITTSBURG.

Read before Pennsylvania State Dental Society, at Glen Summit, July 6, 1997.

Cohesive gold and the control of conditions afforded by the introduction of the rubber-dam made possible large restorations of broken-down tooth-structure. The use of screws in dental operations was born of the necessity for retention in restoring and contouring, and it is to this class that the use of screws is specially applicable. While crowning has taken the place of building up in extreme cases of restoration, the usefulness of screws has not diminished, but has rather increased. By their use foundations may be made for crowns on roots that would otherwise be useless. By the use of small screws, in many cases security of retention can be had where undercuts and grooves would endanger the pulp and throw the strain for retention on structure already greatly weakened. In the minor cases of restoration or contouring where the small screws may be used, the screw does not seem to have had the recognition that it deserves. It may be claimed for it that (if judiciously inserted in those parts of the teeth best able to bear the strain) it gives the maximum retention security with the minimum loss of tooth-structure. The use of the screw, as has been intimated, is in those cases where extra retention security is wanted, and it does not supplant old methods of retention in ordinary cases, though in many it may supplement.

In this model we have assembled a number of typical cases where screws may be used, some where their use would be good practice, and others where their use would be more positively indicated. Taking those where their use would be good practice, we would present first a central incisor with a large cavity on the distal surface; the incisal angle is weak. For filling the cavity as it is, the ordinary methods of retention would be indicated; but if we take into consideration that the incisal angle is liable to fracture, and there may be objections to anticipating this by cutting it away, the resort to the use of screws is certainly good practice, as it will not only afford security for retention as it is, but also in the event of fracture, in which case an additional screw near the incisal edge will give security for retention in restoring the incisal angle.

The upper first bicuspid is another case of this class. The tooth has a filling on the mesial surface extending across the occlusal, including the distal surface. The disto-bucco occlusal angle is contoured. A screw was placed at the disto-bucco gingival angle. The lingual surface is broken off, leaving the filling exposed along the entire lingual side. We have here ample space and room for placing two screws, which will give retention security for restoring the lost part and will assist in retaining the first filling.

The lower first bicuspid, together with the second bicuspid and the first molar, show another class of cases where the small screws may be used to advantage. It is noticeable that the occlusion on the first bicuspid distal surface is close, dipping into the cavity space. This might be filled successfully by undercutting, but with less loss of tooth structure, with greater retention security, and with more ease by the use of a screw. The disto-occlusal angle is wanting, so that a screw can be readily placed pointing toward the mesio-lingual angle.

In the lower second bicuspid the mesio-buccal and lingual angles are broken down; the sulci have no faults; the bite is close; there is a broad seat for the filling at the gingival line. The opposite point of retention, by the ordinary methods, would be made by undercutting near the occlusal surface, or cutting through that surface into the sulci for anchorage. By the use of a screw anchored in the strong body of sound tooth structure, as shown, near the occlusal surface, greater security for retention can be had with no weakening of the tooth structure.

The lower first molar is somewhat similar; in this case the strain being greater, a larger screw may be used. Placing it in the body of the tooth, near the occlusal surface, a seat is made for a small screw near the mesio-bucco-gingival angle, and others might be placed. Another in this class of cases where the small screw may be used to advantage is shown by the tooth in the place of the upper second molar; the tooth is an upper third molar. It is in that tooth, in place, that screws may be used as here shown. Screws are placed through cavity, near the disto-gingival line angle, in the dentine.

Where the breaking down of the angles necessitates a resort to contouring, the use of screws is more positively indicated. The point of opposition for wedging in these cases by the ordinary methods is wanting, unless resort is had to deep cutting, which will endanger the pulp, and even then will not afford the security for retention which can be had by the use of screws. We have here in the model several cases of that class.

There are other cases where the use of screws is positively indicated, where retention security cannot be had without them. The upper cuspid is an example of this class. It is worn down about one half, is perfectly sound; the pulp-chamber is well protected by the secondary dentin. In this case, without disturbing the tooth structure other than for placing screws, perfect retention security can be had. In many cases where access may be had to the pulp canals, screws of the larger size may be used and supplemented by smaller ones. Where roots are badly broken down, so that the margins are wanting, the use of screws for securing a foundation of amalgam for crowning cannot be overestimated.

The location of screws is a matter of considerable importance. It should be at a point readily approached for inserting both the screw and the filling. It should be as far as possible from the pulp and not too near the margin of the tooth, and consequently at a point where there is the greatest bulk of tooth. In the incisors and cuspids the point that meets these requirements best is the linguo-gingival angle, either mesial or distal. This point should be made accessible by free cutting if need be, as it will not weaken the tooth. With caution screws may be placed at the labial angles. Usually it will require considerable space, but there should be no hesitation about making space, as a little more exposure of gold is a matter of small consequence compared with that of avoiding the pulp and margin and getting the screw fixed. Where there is sufficient space a screw may be placed near the incisal edge in the dentin, orherwise there is a liability of encroaching on the enamel plates or the pulp by placing a screw at this point. In the bicuspid and molars where there is sufficient space (and in many cases it would be good practice to make space), the best point is in the center of the body of the tooth, near the occlusal surface. In many cases we have to be governed by the convenience of approach and the relation of the pulp in selecting the point for placing a screw. Where the course of the pulp-canal is used for placing screws, the shape of the roots and their size should be considered, especially in the posterior teeth, where large screws are used. For cavities in the upper molars the lingual root is the best, as it is the largest and most readily approached. For securing a foundation for crowning it is best to put in two screws. This can be done in bicuspids and in molars by using more than one root, In regard to the use of screws in practice, many no doubt "lose the good they oft might win by failing to attempt," and others by failing in the attempt. It would seem to be a simple operation to place a screw, and yet to be successful care must be taken in each detail, and always especially in placing the small screws.

To avoid confusion care should be taken in the selection of the size of the instruments and screws; and where these are not fixed in sets, they should be tested *always* before using. This confusion can in a great measure be avoided by confining ourselves to the use of two or three sizes of screws; in most cases two will do—the small-size anchor screw, 100 threads to the inch; and a large size about forty-eight threads to the inch. For the anchor screw there is a set of instruments complete that could not well be improved upon, though there may be a choice of these. When no drill is furnished with the tap and carrier it is well to test the drill as to size each time before using. As to the manipulation in the use of the anchor screw, having decided where we will place our screw, we should make sure that the way is clear to direct approach to that point. The first step in the manipulation is to countersink with a small round bur a seat for the filling at the point where the drilled hole is to be. With a "limit drill" in the engine, carry the drill steadily up to the shoulder, and then withdraw quickly while it is revolving; if allowed to stop it may break off in the drill-hole when you attempt to remove it. Take the tap, place it cautiously in the drilled hole, give one or two turns; when it is engaged let it be free, so that it may line up with the drilled hole, repeating this procedure with gentle pressure until the tap is arrested. It must be kept in mind that the instrument is a very delicate one and may break in the hole if not handled with great carefulness.

We prefer the screw chuck as a carrier, because we can regulate the length of the screw and the screw is held firm, so that in placing it the thread on the wire or in the tooth is not liable to be torn. With the screw in the carrier the procedure should be the same as with the tap, leaving the instrument free after every few turns, that it may be in a line with the drilled and tapped hole. It should be tested by gentle turning to make sure that it is fixed. Then loosen the handle from the shaft first; unscrew the shaft from the anchor screw. A word of caution: the drill and tap being more slender than the chuck-screw carrier, it must be borne in mind when using the former that room must be left for the use of the latter, so that it may not come in contact with the tooth at any point other than the seat of the filling, when the screw is placed or being placed.

The placing of the larger screws in the pulp-canal while not requiring the same delicacy of manipulation, requires care. Where the roots can be approached directly but with little difficulty will be experienced; where they must be approached at an angle, as in the case of the lower second molar in the model here shown, the main difficulty is in making the drill-hole in a line with the course of the canal. The right-angle attachments do not supply the need. An instrument such as we have here is one that can be depended upon; the reach is long enough and yet not too long; the shifting angle is the main advantage to be had in its use. The short finger chucks can be used for tapping and placing the screws.

The presentation of our subject—the use of the screw for filling, and especially the use of the anchor screw—is not complete without some reference to the manipulation of the screw in filling. The object in making a seat for the filling, in which the screw is placed, is to give stability to the filling. And this is accomplished by condensing the gold around the base of the screw and following it up until the screw is covered. When we consider the limited amount of surface the screw presents, we can understand how necessary it is to condense the gold well to make it secure. For this reason we would advise the use of small pointed pluggers, straight and contracurved. Gold should be worked all around the screw from the first, and not wedged in on one side, leaving the other side unsupported. Some object to the use of screws on account of the complications and care necessary in placing and filling, but this has no weight compared with the security and results attained by their use.—*Dental Cosmos.*

